# Development of an improved preclinical humanized mouse platform representing the diverse clinical phenotypes of Sjögren’s syndrome

**DOI:** 10.3389/fimmu.2026.1793493

**Published:** 2026-05-22

**Authors:** Jin-Sil Park, JeongWon Choi, Ha Yeon Jeong, Hye Yeon Kang, Sang Hee Cho, Su Beom Lee, Mi-La Cho, Sung-Hwan Park

**Affiliations:** 1 Rheumatism Research Center, Catholic Research Institute of Medical Science, College of Medicine, The Catholic University of Korea, Seoul, Republic of Korea; 2 Lab of Translational ImmunoMedicine, Catholic Research Institute of Medical Science, College of Medicine, The Catholic University of Korea, Seoul, Republic of Korea; 3 Department of Pathology, College of Medicine, The Catholic University of Korea, Seoul, Republic of Korea; 4 Department of Medical Sciences, Graduate School of The Catholic University of Korea, Seoul, Republic of Korea; 5 Divisions of Rheumatology, Department of Internal Medicine, Seoul St. Mary’s Hospital, College of Medicine, The Catholic University of Korea, Seoul, Republic of Korea

**Keywords:** CXCR3, humanized mouse, IL-17-producing T cells, NSG mouse, Sjogren’s syndrome

## Abstract

**Background:**

Sjögren’s syndrome (SS) is a systemic autoimmune disease characterized by lymphocytic infiltration of exocrine glands, leading to impaired glandular secretion. To elucidate the pathogenic mechanisms underlying SS, suitable preclinical animal models are essential. In this study, we developed a humanized murine model that captures key immunopathological features of SS patients, and assessed its therapeutic utility.

**Methods:**

PBMCs obtained from SS patients were stimulated with anti-CD3 and anti-CD28 antibodies for 15 hours, and 1 × 10^6^ or 2 × 10^6^ of these cells were intraperitoneally injected into NOD.Cg-*Prkdc* ^scid^ *Il2rg* ^tm1Wjl^/SzJ (NSG) mice. At 5 weeks after cell injection, pathological analysis and immunophenotypic characterization of infiltrating immune cells within the salivary gland tissues were performed. To evaluate the efficacy of metformin, NSG mice transplanted with PBMCs were orally administered metformin daily for 5 weeks.

**Results:**

Mice injected with PBMCs from SS patients exhibited a significant increase in the frequency of human IL-17-producing T cells in the spleen, accompanied by enhanced infiltration of these pro-inflammatory cells into the salivary glands. Histopathological analysis of salivary glands revealed marked immune cell infiltration and a significant reduction in Aquaporin-5 expression in SS-derived PBMC-injected mice. Notably, these pathological changes were associated with the local recruitment of CXCR3+ Th17 cells. Metformin treatment significantly attenuated salivary gland inflammation, reduced the infiltration of pathogenic T cells, and mitigated molecular-level tissue damage in this humanized SS model.

**Conclusions:**

The humanized murine model developed in this study effectively reproduced key cellular and molecular features of SS and provided a useful preclinical platform for investigating early-stage disease mechanisms and evaluating novel therapeutic strategies for SS.

## Introduction

Sjögren’s syndrome (SS) is a systemic autoimmune disorder characterized by reduced glandular secretory function caused by lymphocytic infiltration of the exocrine glands. Destruction of the lacrimal and salivary glands, which typically occurs in patients with SS, leads to keratoconjunctivitis sicca (ocular dryness) and xerostomia (oral dryness) (1). Patients with SS frequently present with extraglandular manifestations, including non-erosive polyarthritis, arthralgias, vasculitis, neuropathy, and chronic fatigue (2, 3). Furthermore, SS is associated with an increased risk of developing various non-Hodgkin lymphomas, which can significantly affect morbidity and mortality (4, 5). Although the etiology and pathogenesis of SS remain incompletely understood, infiltration of T and B lymphocytes into the exocrine glands and their subsequent interactions are considered pivotal mechanisms driving disease development (6, 7).

To elucidate the underlying pathogenesis of SS and to identify potential therapeutic strategies, various genetic and induced animal models have been developed (8). Among these, the non-obese diabetic (NOD) mouse represents a prototypical genetically predisposed model that not only develops type 1 diabetes but also exhibits SS-like autoimmune exocrinopathy, making it widely utilized in SS research (8, 9). Between 8 and 16 weeks of age, NOD mice develop lymphocytic infiltration in the salivary and lacrimal glands, and autoantibodies become detectable in the serum (8). After 16 weeks, salivary flow rates decline significantly (10). Spontaneous mouse strains derived from the NOD background include NOD.B10-H2b and C57BL/6.NOD-Aec1Aec2 (9). In contrast, induced models—generated by exogenous stimuli, such as viral infection or administration of specific antigens—are particularly useful for investigating the early stages of disease pathogenesis. However, only a few disease-associated antigens have been identified to date (9). For instance, in an induced model immunized with subcutaneous injections of mouse submandibular gland extract combined with an adjuvant, lymphocytic infiltration and impaired salivary secretion were observed (11). Similarly, repeated intraperitoneal injections of the Ro peptide, an SS-associated autoantigen, led to decreased salivary secretion and lymphocytic infiltration, although the disease phenotype required repeated administration over more than 5 months to manifest (12, 13). Despite their contributions to the understanding of SS, these models often fail to replicate adequately the complexity of human disease (14). Consequently, there is a growing need for the development of human-mouse chimeric models that enable *in vivo* investigation of SS within a humanized immune context. A foundational study by Young et al. (14) first established a humanized model of SS by transferring human PBMCs from SS patients into immunodeficient NOD scid gamma (NSG) mice (NOD.Cg-*Prkdc*^scid^ *Il2rg*^tm1Wjl^/SzJ), providing critical insights into human immune cell behavior in a murine host. However, further refinements are necessary to enhance the robustness of immune engraftment and better recapitulate the multifaceted clinical pathology of SS.

In this study, we established a refined preclinical mouse model of SS by intraperitoneally injecting peripheral blood mononuclear cells (PBMCs) from patients with SS into NSG mice. To optimize the pathogenicity and engraftment of the human cells, we implemented a methodological advancement by pre-stimulating the PBMCs with anti-CD3 and anti-CD28 Antibodies prior to injection. This enhanced humanized murine model of SS exhibited increased infiltration of cells expressing inflammatory cytokines and C-X-C motif chemokine ligand (CXCL)9 or CXCL10 within the salivary glands, consistent with pathological features observed in human SS. Additionally, an elevated number of CXC chemokine receptor 3 (CXCR3)+ Th17 cells were detected in the salivary gland. Furthermore, we utilized this improved platform to validate the therapeutic efficacy of metformin, which significantly alleviated salivary gland inflammation and tissue damage. These results demonstrate that our refined humanized model serves as a potent and reliable tool for evaluating novel therapeutic strategies in SS.

## Materials and methods

### SS patients and PBMC isolation

Peripheral blood samples were obtained from healthy controls (HC, n = 5) and SS patients (n = 6) who visited Seoul St. Mary’s Hospital, a tertiary referral center in the Republic of Korea (KC17TNSI0237). The clinical baseline characteristics of the study participants, including age, sex, autoantibody titers, and ESSDAI scores, are summarized in **Supplementary Table 1**. PBMCs were isolated from heparinized blood using Ficoll-Paque density gradient centrifugation, following standard protocols (15).

### Mice and induction of the humanized model

Six- to eight-week-old female NSG mice were purchased from The Jackson Laboratory (Bar Harbor, ME, USA). The animals were housed under specific pathogen-free conditions at the Institute of Medical Science, Catholic University of Korea, and were provided with standard chow and water ad libitum. All animal procedures were approved by the Animal Research Ethics Committee of the Catholic University of Korea and conducted in accordance with the United States National Institutes of Health guidelines (permit numbers: CUMS-2014-0123-02; 2017-0066-04). All mice were treated and euthanized following the Guidelines on the Use and Care of Animals of the Catholic University of Korea. Surgical procedures were performed under isoflurane anesthesia, and all efforts were made to minimize animal suffering. At the conclusion of the experiment, the mice were euthanized in a CO_2_ chamber for tissue collection.

To establish the humanized SS model, PBMCs isolated from the blood of healthy controls and SS patients were stimulated with anti-CD3 antibody (1 µg/mL) and anti-CD28 antibody (1 µg/mL) for 15 hours. For initial model characterization, 1 × 10^6^ or 2 × 10^6^ activated PBMCs were intraperitoneally injected into 7-week-old female NSG mice. For the subsequent therapeutic efficacy studies, the PBMC dose was standardized to 1 × 10^6^ cells per mouse to ensure a uniform and reproducible inflammatory environment across all experimental groups. Metformin (50 mg/kg; Sigma-Aldrich) dissolved in saline was administered orally once daily for five consecutive weeks following PBMC infusion. Saliva secretion was stimulated in anesthetized mice by intraperitoneal injection of pilocarpine (Sigma-Aldrich, St. Louis, MO, USA) at a dose of 5 mg/kg body weight. Saliva was collected from the oral cavity for 7 min, beginning 90 s after the injection, using a micropipette. The volume of saliva was determined gravimetrically (μL/g/min). Each *in vivo* experiment was performed using PBMCs from multiple independent donors (Total 5 HCs and 6 SS patients) to ensure biological diversity, with each mouse treated as an independent biological replicate (n).

### Intracellular staining and flow cytometry

For surface marker staining, single-cell suspensions were washed with fluorescence-activated cell sorting buffer (phosphate-buffered saline containing 2% fetal bovine serum) and incubated with fluorochrome-conjugated antibodies for 30 minutes at 4 °C. For intracellular staining, single-cell suspensions were stimulated with 25 ng/mL phorbol myristate acetate (Sigma-Aldrich) and 250 ng/mL ionomycin (Sigma-Aldrich) in the presence of GolgiStop (BD Biosciences) for 4 hours. Following surface staining, cells were fixed and permeabilized using a Cytofix/Cytoperm kit according to the manufacturer’s instructions (BD Biosciences). For intracellular Foxp3 staining, a Foxp3/Transcription Factor Staining Buffer Kit (eBioscience) was used after surface staining. Cells were then washed with Perm/Wash buffer, incubated with the respective intracellular antibodies for 30 minutes at 4 °C, and analyzed through flow cytometry. The following anti-human antibodies were used: PE/Cyanine7 anti-human CD4 Antibody (RPA-T4, BioLegend, #300511), Alexa Fluor^®^ 647 Mouse anti-Human IL-17A (SCPL1362, BD Biosciences, #560437), and FITC anti-human Foxp3 (PCH101, eBioscience, #11-4776-42). Stained cells were analyzed using a CytoFLEX (Beckman Coulter) or LSRII (BD Biosciences) flow cytometer. Data were acquired and analyzed using FlowJo software (Tree Star).

### Histological analysis

Salivary gland tissues were fixed in 10% (v/v) neutral buffered formalin (Sigma-Aldrich) and embedded in paraffin. Sections (5 µm thick) were prepared and stained with hematoxylin and eosin for general histological evaluation. For immunohistochemistry, tissue sections were processed using a Dako REAL EnVision Detection Systems kit (DAKO, Glostrup, Denmark, #5007). Sections were incubated overnight at 4 °C with primary antibodies against CD4, IL-17, IL-6, TNF-α, aquaporin-5 (AQP5), Caspase-3, CXCL9, and CXCL10, followed by incubation for 30 minutes with a horseradish peroxidase-conjugated secondary antibody. Visualization was performed using the chromogen diaminobenzidine. Stained tissues were examined by photomicroscopy (Olympus), and the percentage of the stained area was quantified using ImageJ software from high-power digital images (400× magnification). For confocal microscopy, sections were incubated overnight at 4 °C with primary antibodies against CD4, IL-17, and CXCR3. After washing, secondary antibodies conjugated with FITC, PE, or APC were applied for 1 hour at room temperature. Nuclei were counterstained with 4′,6-diamidino-2-phenylindole. Confocal images were acquired at 400× magnification using an LSM 800 confocal microscope (Zeiss, Oberkochen, Germany).

### Statistical analysis

All statistical analyses were performed using GraphPad Prism (v.10 for Windows; GraphPad Software, Inc., La Jolla, CA, USA). *In vivo* experiments were conducted using PBMCs from 5 healthy donors and 6 SS patients in total. Specifically, 4 HCs and 4 SS patients were assigned to the model characterization phase, and 3 HCs and 3 SS patients were assigned to the metformin treatment phase, with some donors contributing to both phases to ensure inter-experimental reproducibility. Each mouse was treated as an independent biological replicate (n). For comparisons between two groups, a two-tailed unpaired Student’s t-test was used. For comparisons involving three or more groups, a one-way analysis of variance (ANOVA) followed by Tukey’s *post-hoc* test was performed. For longitudinal data, a two0way ANOCA was applied. Data are presented as means ± standard error of the mean (SEM). *P*-values < 0.05 (two-tailed) were considered statistically significant.

## Results

### Increased frequency of splenic Th17 cells and salivary gland inflammation in humanized mice derived from PBMCs of SS patients

**Figure 1:**
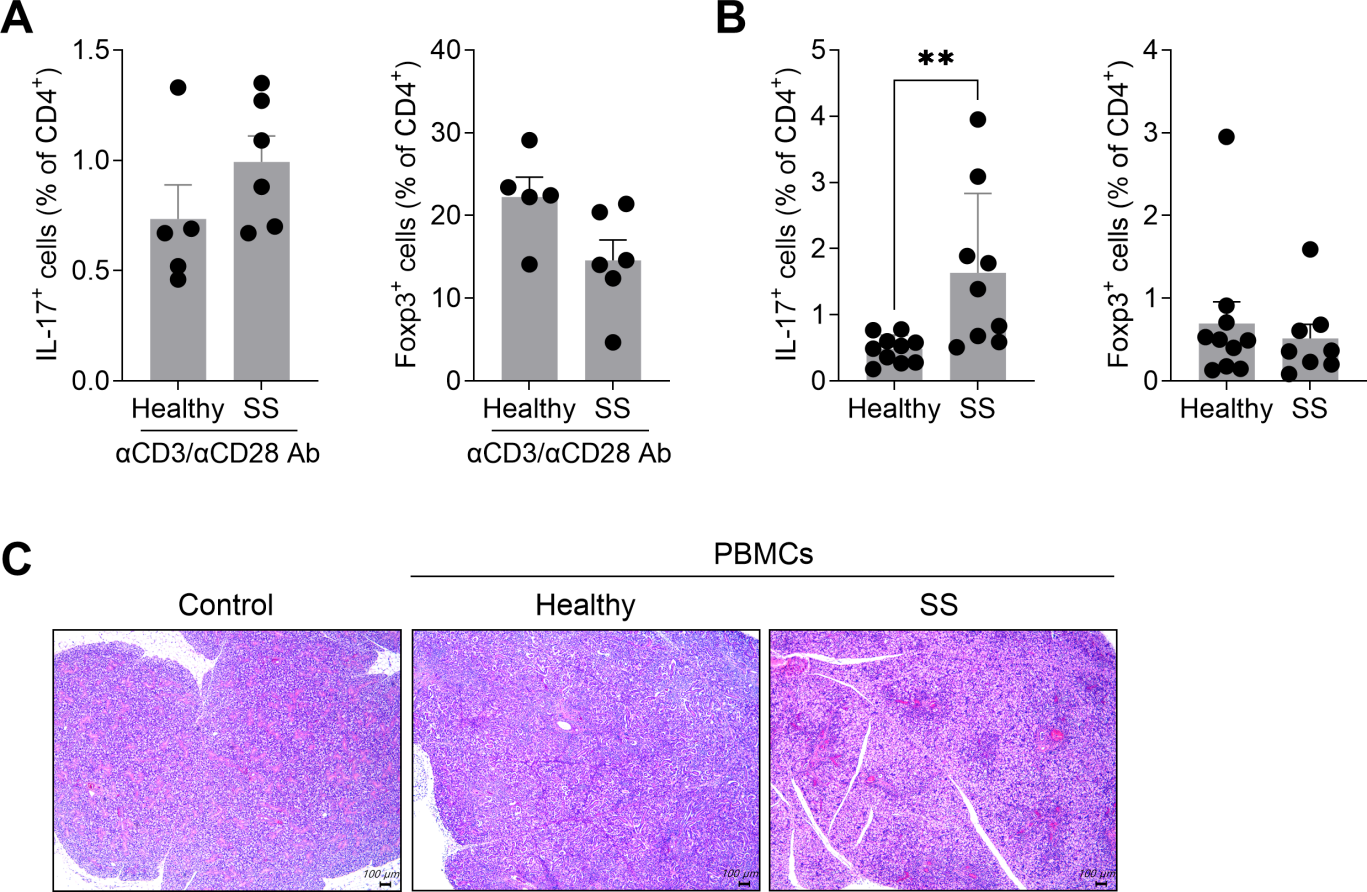
Establishment of a humanized murine model of SS using human PBMCs stimulated with anti-CD3 and anti-CD28 antibodies. PBMCs isolated from the blood of healthy donors and SS patients were stimulated with anti-CD3 antibody (1 μg/mL) and anti-CD28 antibody (1 μg/mL) for 15 hours. **(A)** The frequencies of human CD4+IL-17+ and CD4+Foxp3+ cells were analyzed by flow cytometry prior to injection. Each symbol represents an independent donor (Healthy, n = 5, SS, n = 6). **(B)** In total, 2 × 10^6^ of these cells were intraperitoneally injected into 7-week-old female NSG mice. Five weeks after PBMC injection, the frequencies of human CD4+IL-17+ and CD4+Foxp3+ cells in murine splenocytes were assessed by flow cytometry. **(C)** At 5 weeks after PBMC injection, murine salivary gland tissues were stained with hematoxylin and eosin. Representative H&E staining of salivary gland section. Each symbol represents an independent donor **(A)** or an individual mouse **(B)**. For splenocyte analysis **(B)**, the final n (Healthy, n = 10; SS, n = 9). Data expressed as mean ± SEM. **P < 0.01 (unpaired Student’s *t*-test). Data are representative of two independent experiments using a total of 4 healthy donors and 4 SS donors.

To establish a humanized SS mouse model, NSG mice were intraperitoneally injected with PBMCs obtained from patients with SS. PBMCs isolated from healthy individuals and SS patients were first stimulated with anti-CD3 and anti-CD28 antibodies, and their phenotypic profiles were analyzed by flow cytometry after 15 hours of culture. Compared to PBMCs from HCs, those from SS patients exhibited a markedly higher frequency of IL-17+CD4+ cells and a lower frequency of CD4+Foxp3+ cells (**Figure 1A**). These activated PBMCs were then injected into NSG mice to induce a humanized SS model. Five weeks following PBMC injection, the frequency of immune cell subsets in the spleen was analyzed. Mice injected with PBMCs from SS patients displayed a significant increase in splenic CD4+IL-17+ cell populations compared with mice injected with PBMCs from healthy controls, while no significant difference was observed in the frequency of CD4+Foxp3+ cells (**Figure 1B**). Additionally, histological examination revealed a greater degree of lymphocytic infiltration in the salivary gland tissues of mice injected with SS patient-derived PBMCs compared with those receiving PBMCs from healthy individuals (**Figure 1C**). Collectively, these findings demonstrated the successful establishment of a humanized mouse model that recapitulates key immunological features of SS.

### Enhanced inflammatory responses in the salivary gland tissue of humanized mice injected with PBMCs from SS patients

**Figure 2:**
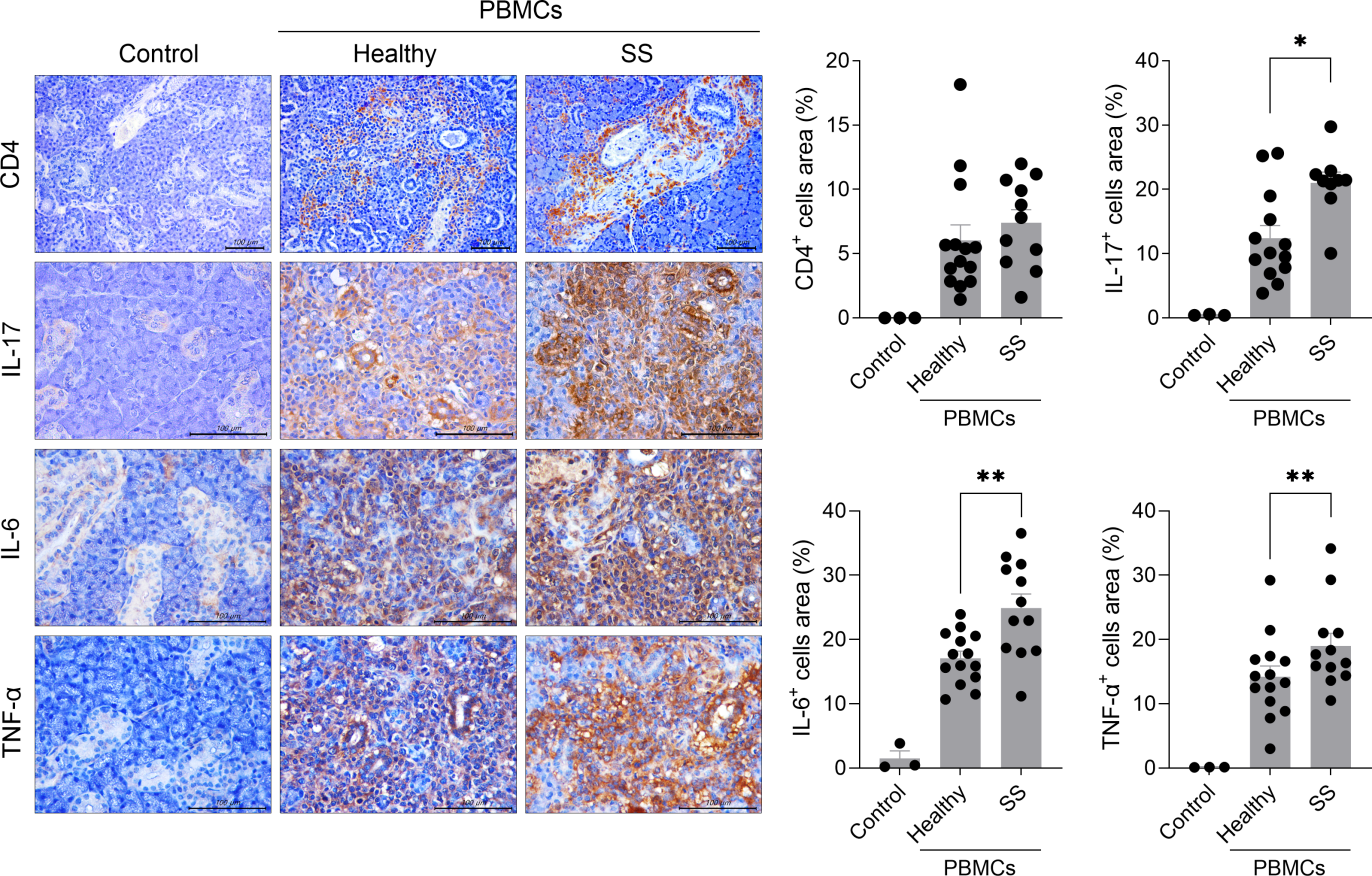
Transplanted PBMCs from SS patients promote inflammation in the salivary gland tissue of immunodeficient mice. PBMCs isolated from the blood of healthy donors and SS patients were stimulated with anti-CD3 antibody (1 μg/mL) and anti-CD28 antibody (1 μg/mL) for 15 hours. In total, 2 × 10^6^ of these cells were intraperitoneally injected into 7-week-old female NSG mice. At 5 weeks after PBMC injection, salivary glands were harvested from humanized mice. Sections were subjected to immunohistochemical staining for human CD4, IL-17, IL-6, and TNF-α. Representative histological features are shown. The graph depicts the areas positive for CD4+, IL-17+, IL-6+, or TNF-α+ cells. Magnification, 200×; scale bar, 100 μm. Each symbol represents an individual mouse (Healthy, n = 14; SS, n = 12). Data expressed as mean ± SEM. *P < 0.05, **P < 0.01 (unpaired Student’s *t*-test). Data are representative of two independent experiments utilizing a total of 4 healthy donors and 4 SS donors.

Next, we investigated whether salivary gland inflammation was promoted in the humanized SS model induced by PBMCs from SS patients. Consistent with the systemic increase in human Th17 cells observed in the spleen (**Figure 1B**), similar degrees of CD4+ T-cell infiltration were observed in the salivary glands of both healthy PBMC-injected and SS PBMC-injected mice. However, compared with the healthy PBMC group, the mice injected with SS patient-derived PBMCs showed a significantly higher infiltration of IL-17-producing cells (*P* < 0.05) and IL-6-producing cells (*P* < 0.01) within the salivary gland tissue. Although there was a trend toward increased infiltration of TNF-α-producing cells in SS PBMC-injected mice, this difference did not reach statistical significance (**Figure 2**). These results indicate that PBMCs from SS patients promoted a potent pro-inflammatory environment within the salivary glands of humanized mice.

### Exacerbation of salivary gland tissue damage in humanized mice injected with PBMCs from SS patients

**Figure 3:**
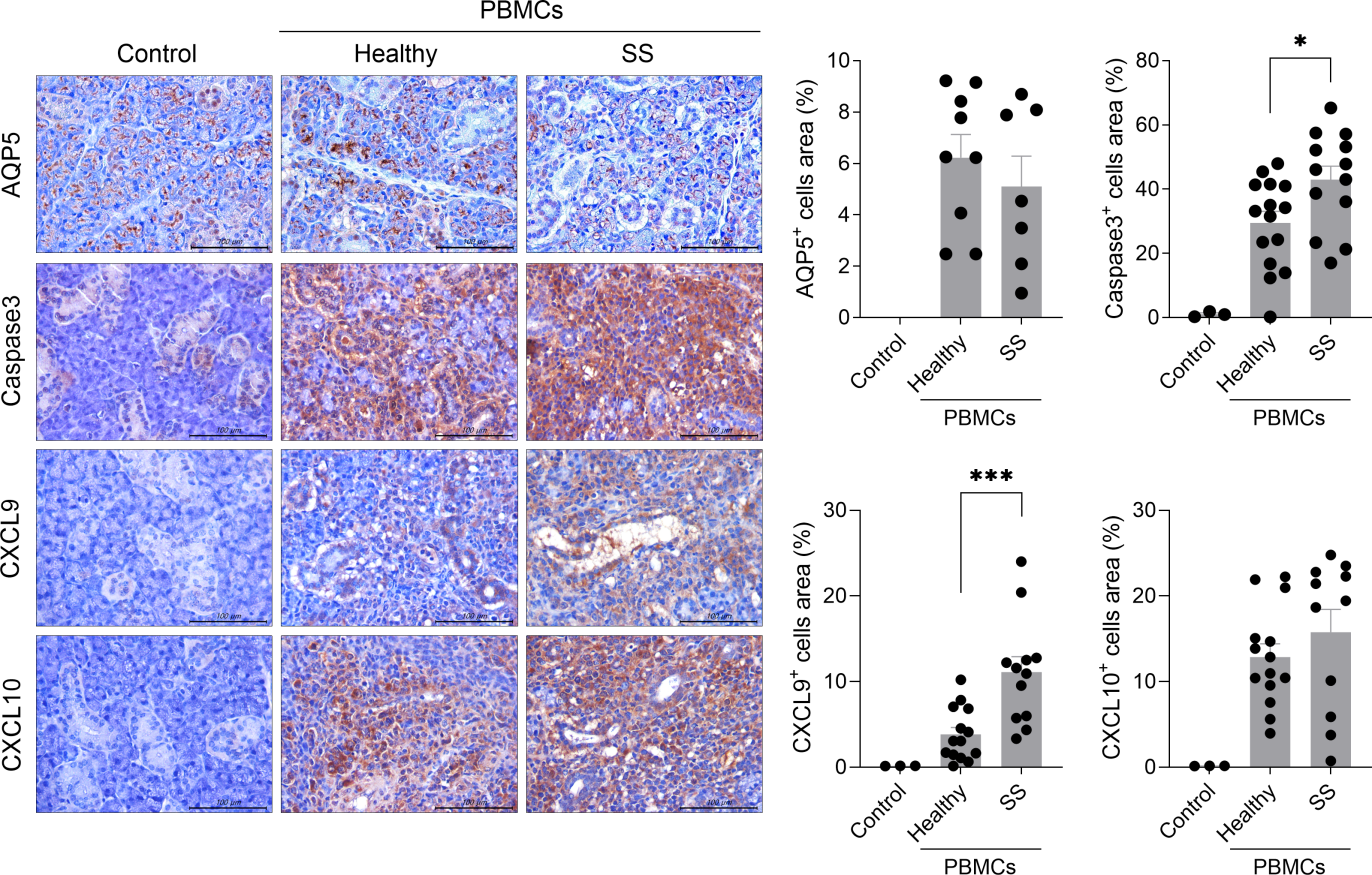
Transplanted PBMCs from SS patients exacerbate salivary gland tissue damage in immunodeficient mice. PBMCs isolated from the blood of healthy donors and SS patients were stimulated with anti-CD3 antibody (1 μg/mL) and anti-CD28 antibody (1 μg/mL) for 15 hours. In total, 2 × 10^6^ of these cells were intraperitoneally injected into 7-week-old female NSG mice. At 5 weeks after PBMC injection, salivary glands were harvested from humanized mice. Sections were subjected to immunohistochemical staining for human AQP5, caspase-3, CXCL9, and CXCL10. Representative histological features are shown. The graph indicates the areas positive for human AQP5+, caspase-3+, CXCL9+, and CXCL10+ cells. Magnification, 200×; scale bar, 100 μm. Each symbol represents an individual mouse (Healthy, n = 8-16; SS, n = 8-12). Data expressed as mean ± SEM. *P < 0.05, ***P < 0.001 (unpaired Student’s *t*-test). Data are representative of two independent experiments utilizing a total of 4 healthy donors and 4 SS donors.

To assess salivary gland function and tissue integrity, the expression of key factors involved in glandular homeostasis was evaluated. Compared to mice injected with healthy PBMCs, those injected with PBMCs from SS patients exhibited a significant reduction in AQP5-expressing cells, a water channel protein that is primarily localized in the apical membrane of salivary gland acinar cells and is critical for saliva secretion (16). Consistent with findings in SS patients, in whom caspase-3 activation is abnormally elevated in salivary epithelial cells (17), a significant increase in caspase-3-positive cells was detected in the salivary gland tissues of SS humanized mice relative to controls. Furthermore, given that CXCL9 and CXCL10 are inflammatory chemokines that regulate immune cell migration, differentiation, and activation (18), their expression was analyzed in the salivary glands. The infiltration of CXCL9-expressing cells was significantly higher in SS humanized mice compared with controls, and CXCL10-expressing cells also showed a tendency toward increased infiltration (**Figure 3**).

### Increased numbers of CXCR3+ Th17 cells in the salivary gland of humanized mice injected with PBMCs from SS patients

**Figure 4:**
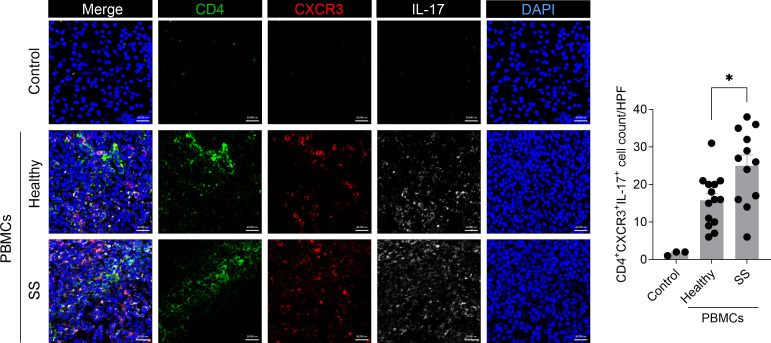
Increased numbers of human CXCR3+ Th17 cells in the salivary gland tissue of immunodeficient mice injected with PBMCs from SS patients. PBMCs isolated from the blood of healthy donors and SS patients were stimulated with anti-CD3 antibody (1 μg/mL) and anti-CD28 antibody (1 μg/mL) for 15 hours. In total, 2 × 10^6^ of these cells were intraperitoneally injected into 7-week-old female NSG mice. At 5 weeks after PBMC injection, salivary glands were harvested from humanized mice. Immunofluorescence staining was performed on salivary gland sections using human anti-CD4 (green), anti-IL-17 (white), and anti-CXCR3 (red) antibodies. Magnification, 400×; scale bar, 20 μm. Representative images and quantification of human CD4+IL-17+CXCR3+ cells manually counted at high magnification are shown. Each symbol represents an individual mouse (Healthy, n = 14; SS, n = 12). Data expressed as mean ± SEM. *P < 0.05 (unpaired Student’s *t*-test). Data are representative of two independent experiments utilizing a total of 4 healthy donors and 4 SS donors.

CXCR3 facilitates T-cell migration to inflamed tissues through its interaction with the chemokine ligands CXCL9, CXCL10, and CXCL11 (19). This receptor is expressed on a subset of Th17 cells and regulates their migration in autoimmune and inflammatory diseases, including non-alcoholic fatty liver disease (20, 21). In the present study, compared with the control group, a significant increase in the numbers of CD4+IL-17+ cells expressing CXCR3 was observed in the salivary gland of humanized mice injected with PBMCs from SS patients (**Figure 4**). These findings imply that elevated numbers of CXCR3+ pathogenic Th17 cells in the inflamed salivary gland of humanized SS mice may promote their migration toward salivary gland tissues with increased CXCL9 and CXCL10 expression (**Figure 4**), thereby contributing to salivary gland inflammation and tissue damage.

### Metformin administration alleviates salivary gland inflammation in humanized SS mice

**Figure 5:**
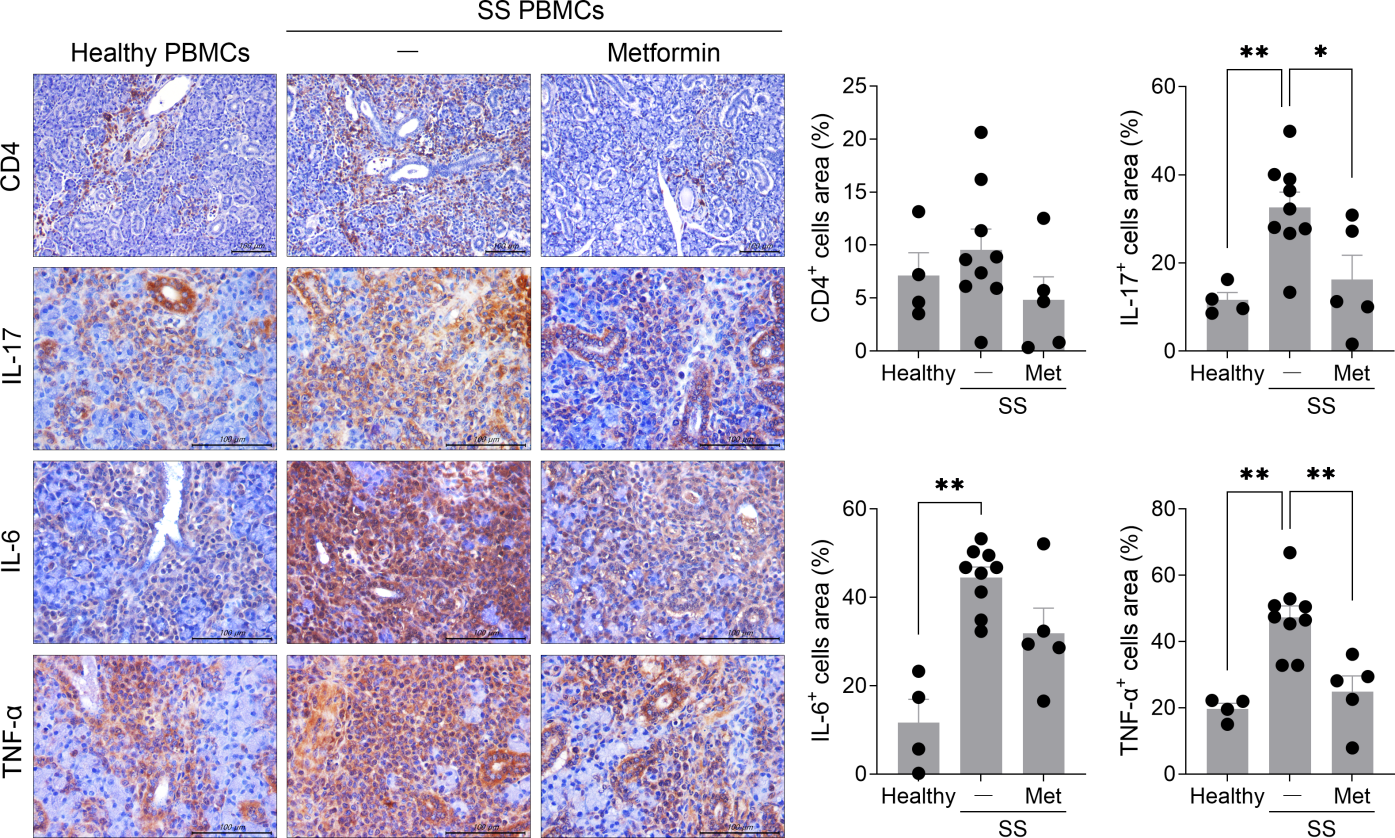
Metformin treatment ameliorates salivary gland inflammation in a humanized murine model transplanted with PBMCs from SS patients. PBMCs isolated from the blood of SS patients were stimulated with anti-CD3 antibody (1 μg/mL) and anti-CD28 antibody (1 μg/mL) for 15 hours. In total, 1 × 10^6^ of these cells were intraperitoneally injected into 7-week-old female NSG mice, and metformin (50 mg/kg) was administered orally once daily for 5 weeks. Based on the initial characterization, a standardized dose of 1 × 10^6^ PBMCs was used to evaluate therapeutic efficacy. **(A)** At 5 weeks after PBMC injection, murine salivary gland tissues were stained with hematoxylin and eosin. **(B)** Salivary gland sections were subjected to immunohistochemical staining for human CD4, IL-17, IL-6, and TNF-α. Representative histological features are shown. The graphs depict the areas positive for human CD4+, IL-17+, IL-6+, or TNF-α+ cells. Magnification, 200×; scale bar, 100 μm. Each symbol represents an individual mouse (Healthy, n = 5; SS + vehicle, n = 9; SS + Metformin, n = 5). Data expressed as mean ± SEM. *P < 0.05, **P < 0.01 (One-way ANOVA followed by Tukey’s *post-hoc* test). Data are representative of two independent experiments utilizing a total of 3 healthy donors and 3 SS donors.

In a previous study, metformin was shown to restore salivary flow and reduce salivary gland inflammation in NOD mice (22). To assess whether metformin exerts similar therapeutic effects in the humanized SS model, metformin was administered orally on a daily basis for five weeks to NSG mice injected with PBMCs from SS patients, after which salivary gland inflammation was evaluated. Compared to the control group, humanized SS mice showed significantly increased lymphocytic infiltration in the salivary glands, which was significantly alleviated by metformin treatment (**Figure 5A**). Furthermore, metformin-treated mice displayed a trend toward reduced CD4+ T-cell infiltration compared to untreated SS humanized mice. Consistent with previous findings, mice injected with PBMCs from SS patients exhibited a significant increase in the infiltration of IL-17-, IL-6-, and TNF-α-expressing cells in the salivary gland tissues compared with healthy PBMC-injected controls. Metformin administration significantly reduced the infiltration of IL-17- and TNF-α-expressing cells and tended to decrease IL-6-expressing cell infiltration in the salivary glands of SS humanized mice. These findings demonstrate that the humanized SS model effectively recapitulates the inflammation seen in patients and can be used to evaluate the therapeutic efficacy of anti-inflammatory agents, such as metformin (**Figure 5B**). To evaluate the functional effects of metformin, salivary flow rate (SFR) was monitored during a 5-week observation period. No statistically significant decrease in SFR was observed in the untreated SS-negative group compared to the healthy PBMC-injected controls, but the metformin treatment group showed a tendency to maintain SFR at week 5 (**Supplementary Figure 1**). This functional trend, while not statistically significant, aligns with the preservation of molecular markers such as AQP5, suggesting that metformin may delay the progression of secretory dysfunction by alleviating early-stage glandular inflammation.

### Metformin administration ameliorates salivary gland tissue damage in humanized mice derived from SS patient PBMCs

**Figure 6:**
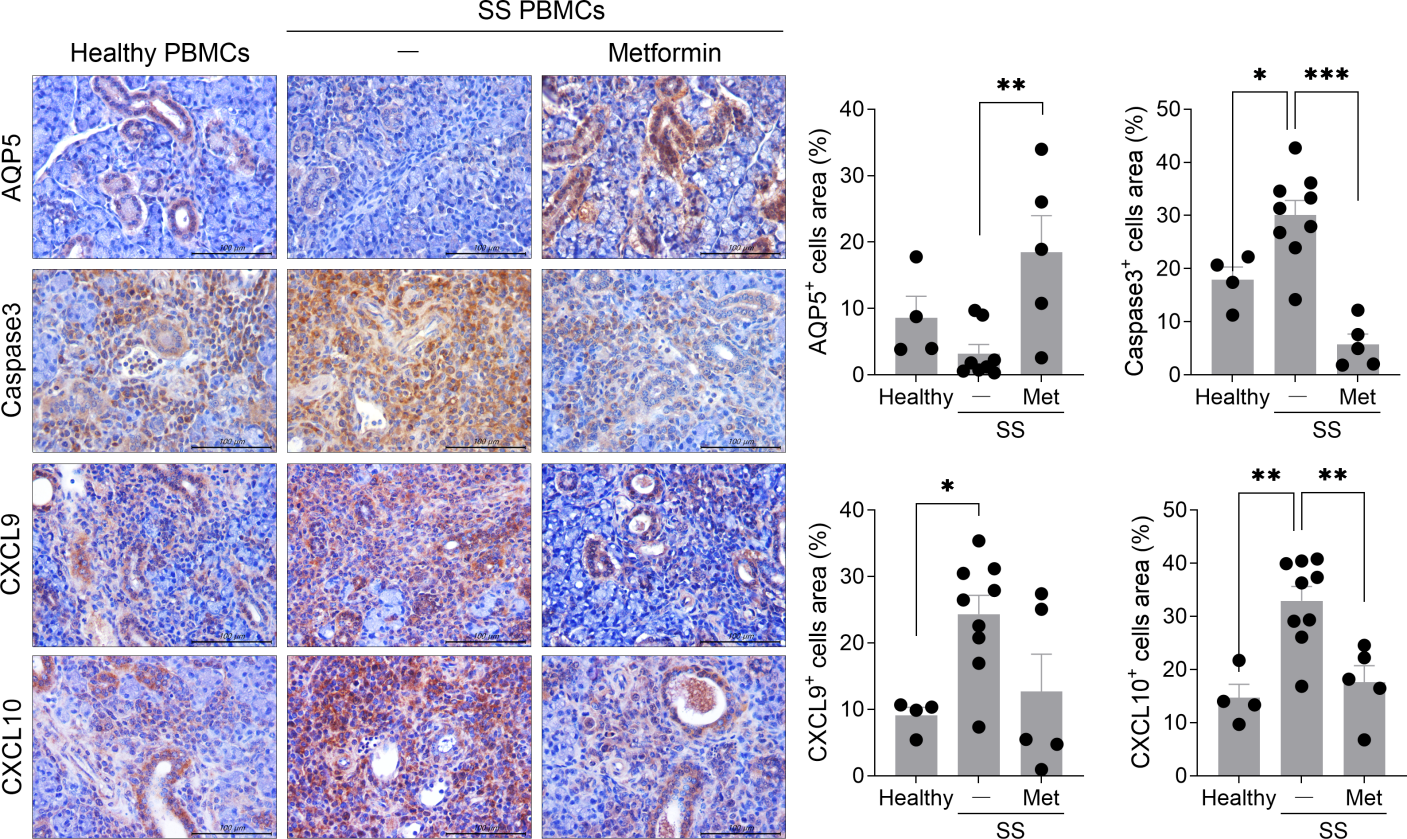
Metformin treatment reduces salivary gland tissue damage in a humanized murine model transplanted with PBMCs from SS patients. PBMCs isolated from the blood of SS patients were stimulated with anti-CD3 antibody (1 μg/mL) and anti-CD28 antibody (1 μg/mL) for 15 hours. In total, 1 × 10^6^ of these cells were intraperitoneally injected into 7-week-old female NSG mice, and metformin (50 mg/kg) was administered orally once daily for 5 weeks. At 5 weeks after PBMC injection, salivary glands were harvested from humanized mice. Sections were subjected to immunohistochemical staining for human AQP5, caspase-3, CXCL9, and CXCL10. Representative histological features are shown. The graphs indicate the areas positive for human AQP5+, caspase-3+, CXCL9+, and CXCL10+ cells. Magnification, 200×; scale bar, 100 μm. Each symbol represents an individual mouse (Healthy, n = 5; SS + vehicle, n = 9; SS + Metformin, n = 5). Data expressed as mean ± SEM. *P < 0.05, **P < 0.01, ***P < 0.001 (One-way ANOVA followed by Tukey’s *post-hoc* test). Data are representative of two independent experiments utilizing a total of 3 healthy donors and 3 SS donors.

To assess further the impact of metformin on salivary gland tissue integrity, histological and immunohistochemical analyses were performed in humanized mice derived from SS patient PBMCs. Metformin administration significantly increased the distribution of AQP5-expressing cells in the salivary gland tissue, implying a potential for the preservation of glandular secretory function at the molecular level. In contrast, metformin significantly reduced the distribution of cells expressing activated caspase-3, indicating decreased epithelial cell apoptosis. Moreover, metformin treatment tended to reduce the infiltration of CXCL9-expressing cells and significantly decreased the infiltration of CXCL10-expressing cells within salivary gland tissues (**Figure 6**). Collectively, these findings demonstrate that metformin attenuates salivary gland tissue injury by modulating apoptosis and chemokine-mediated inflammation in humanized SS mice.

### Metformin administration reduces the number of CXCR3+ Th17 cells in the salivary gland of humanized mice derived from SS patient PBMCs

**Figure 7:**
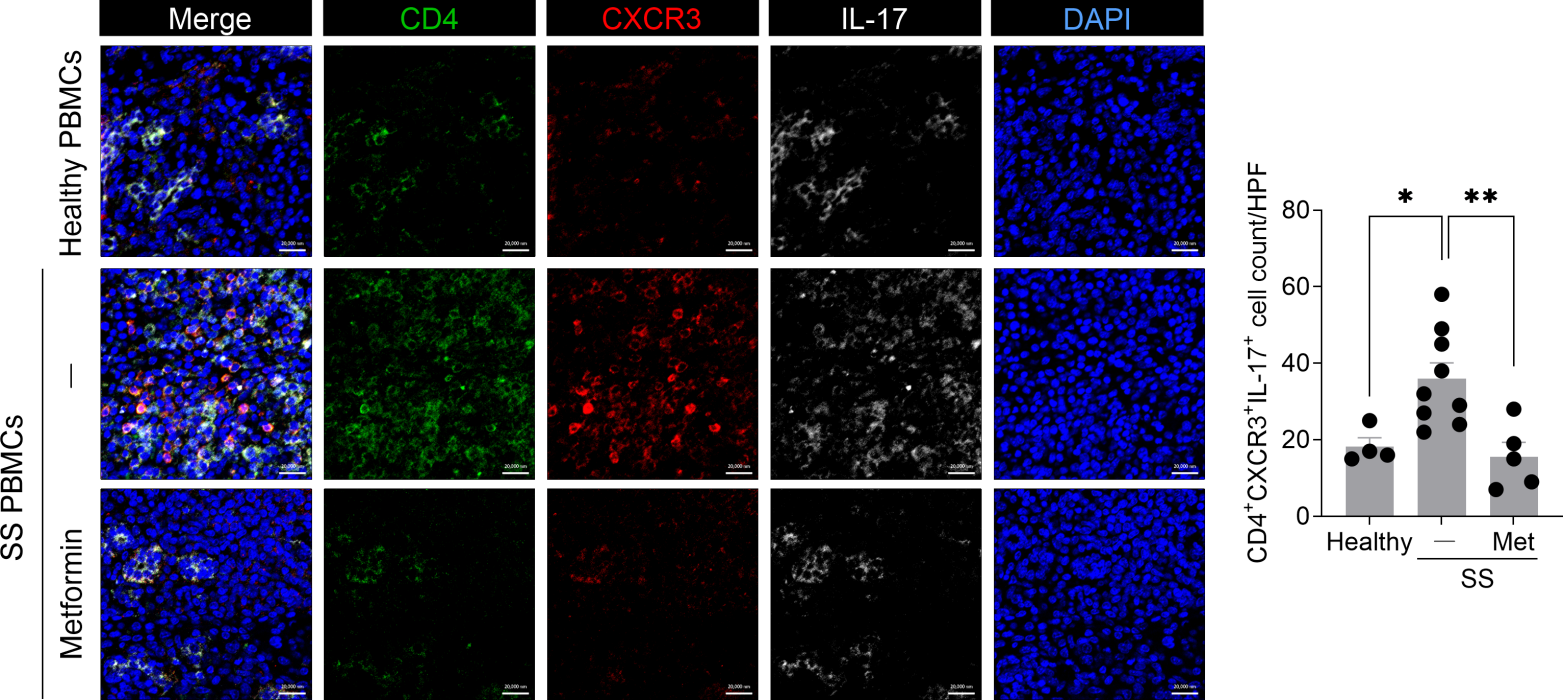
Metformin treatment reduces the number of human CXCR3+ Th17 cells in the salivary gland of a humanized murine model transplanted with PBMCs from SS patients. PBMCs isolated from the blood of SS patients were stimulated with anti-CD3 antibody (1 μg/mL) and anti-CD28 antibody (1 μg/mL) for 15 hours. In total, 1 × 10^6^ of these cells were intraperitoneally injected into 7-week-old female NSG mice, and metformin (50 mg/kg) was administered orally once daily for 5 weeks. Salivary gland sections were subjected to immunofluorescence staining with human anti-CD4 (green), anti-IL-17 (white), and anti-CXCR3 (red) antibodies. Magnification, 400×; scale bar, 20 μm. Representative images and quantitative graphs showing the numbers of human CD4+IL-17+CXCR3+ cells manually counted at high magnification are presented. Each symbol represents an individual mouse (Healthy, n = 5; SS + vehicle, n = 9; SS + Metformin, n = 5). Data expressed as mean ± SEM. *P < 0.05, **P < 0.01 (One-way ANOVA followed by Tukey’s *post-hoc* test). Data are representative of two independent experiments utilizing a total of 3 healthy donors and 3 SS donors.

To investigate whether metformin modulates the recruitment of pathogenic T cells to the target organ, we performed multi-color immunofluorescence analysis on salivary gland tissues. Compared to humanized mice generated from healthy PBMCs, those derived from SS patient PBMCs exhibited a significant increase in the numbers of CXCR3+CD4+IL-17+ cells in the salivary gland. Importantly, metformin administration markedly reduced the numbers of salivary gland-infiltrating CD4+IL-17+ cells expressing CXCR3 in the SS humanized mice (**Figure 7**). These results imply that metformin exerts its therapeutic effects, at least in part, by directly limiting the recruitment and accumulation of CXCR3+ Th17 cells in the inflamed salivary gland tissue. In summary, while our 5-week observation period captures an early immunopathological phase rather than a full clinical rescue of secretory function, these results consistently demonstrate that metformin mitigates the hallmark molecular and cellular features of SS. Our findings suggest that this humanized platform is a viable preclinical tool for investigating early disease-modifying therapies, such as metformin, that target the Th17-CXCR3 axis.

## Discussion

In this study, we established a humanized SS mouse model that reproduces the salivary gland inflammation and tissue damage observed in SS patients by injecting PBMCs from SS patients into immunodeficient NSG mice. Injection of SS patient-derived PBMCs led to increased infiltration of IL-17+ and IL-6+ cells into salivary gland tissue, accompanied by a decrease in cells expressing the water channel protein AQP5. Moreover, PBMC injection elevated the numbers of cells expressing CXCL9 and CXCL10, chemokines that bind to CXCR3-expressing cells and promote their migration to inflammatory sites. This increase coincided with a higher frequency of CXCR3-expressing CD4+IL-17+ cells in the salivary gland. Within this model, metformin demonstrated the potential to attenuate these hallmark pathological features of SS at the molecular and cellular levels.

Immunodeficient NSG mice are a transgenic strain that lacks functional T cells, B cells, and natural killer cells, and are therefore capable of efficiently engrafting human CD34+ hematopoietic stem cells, PBMCs, or adult stem cells and tissues (14, 23). Human PBMC engraftment was reproducibly maintained at 4 weeks after injection of 20 × 10^6^ PBMCs into NSG mice, without the occurrence of graft-versus-host disease symptoms (24). This represents a substantially lower PBMC dose compared with NOD-scid mice, which require injection of 50 × 10^6^ to 300 × 10^6^ PBMCs to achieve stable engraftment (25). Young et al. administered 5 × 10^6^ freshly isolated human PBMCs intraperitoneally to NSG mice and observed the presence of CD4 and CD8 T cells in the blood after 4 weeks, along with elevated serum levels of inflammatory cytokines such as IFN-γ, IL-17, IL-6, and TNF-α. PBMCs from SS patients, compared to those from healthy donors, induced marked inflammation in the lacrimal and salivary glands, with predominant infiltration of CD4+ T cells (14). Similarly, Yang et al. adoptively transferred 10 × 10^6^ PBMCs into NSG mice and induced disease through subcutaneous immunization with salivary gland proteins from wild-type C57/6J mice. In this model, the mice that received PBMCs from SS patients exhibited significantly reduced salivary flow rates and decreased expression of salivary secretion-related genes, including AQP4, AQP5, and muscarinic acetylcholine receptor M3, compared to mice receiving PBMCs from healthy donors. Furthermore, these mice demonstrated upregulation of interferon-related genes and apoptosis-associated factors that are critical in SS pathogenesis (23).

In this study, the 5-week observation period following PBMC infusion was strategically determined to strike a balance between the expression of SS-specific pathology and the prevention of systemic graft-versus-host disease (GVHD). In humanized NSG models, prolonged observation exceeding 6–8 weeks can lead to lethal GVHD, potentially distorting the interpretation of organ-specific autoimmune responses. By focusing on this 5-week critical period for assessment, we captured the peak influx of human immune cells and the subsequent onset of glandular damage without interference from systemic inflammation. We observed that human T-cell transplantation in NSG mice stabilized within the first 2 weeks, followed by gradual infiltration into exocrine glands. By analyzing the mice at week 5, we were able to capture an established inflammatory phase characterized by CXCR3+ Th17 cell recruitment (**Figures 4**, **7**) and AQP5 degradation (**Figures 3**, **6**), while simultaneously preemptively excluding systemic disturbances of GVHD, which typically worsen after 6 weeks in this xenograft system.

While existing xenograft models require a large number of PBMCs (up to 20-50 × 10^6^ cells) for successful transplantation, the model developed in this study demonstrates that significant SS-like pathology can be induced reproducibly with a much smaller cell volume (1-2 × 10^6^ cells) by pre-activating PBMCs with anti-CD3/CD28 antibodies. This optimization ensures the efficient use of limited patient-derived PBMCs while providing a consistent baseline for evaluating therapeutic modulation. This methodological divergence from the foundational study by Young et al. (14) represents a significant refinement in the humanized model of SS. While Young et al. utilized freshly isolated, resting PBMCs to observe spontaneous immune responses, our approach involves T cell receptor pre-activation for 15 hours prior to engraftment. The rationale for this pre-stimulation is based on the critical role of the CXCL9/10-CXCR3 axis in SS pathogenesis. According to previous report, resting human T cells express negligible levels of CXCR3; however, TCR stimulation significantly induces CXCR3 expression and synergistically enhances T cell proliferation and migratory capacity (19). By pre-activating PBMCs, we pre-emptively induced these chemokine receptors and inflammatory cytokines, thereby maximizing the targeted infiltration of human T cells into the salivary glands. Consequently, this pre-activation strategy specifically accelerates and reinforces the CXCL9/10-CXCR3-mediated salivary gland infiltration, allowing for a successful recapitulation of SS pathology within the optimized 5-week observation period mentioned above. Despite the substantially reduced cell numbers, our model effectively reproduced key pathological features of SS. Compared with the healthy PBMC-injected group, the mice injected with SS patients’ PBMCs exhibited comparable CD4+ T cell infiltration in the salivary glands but showed significantly increased infiltration of IL-17+, IL-6+, and TNF-α+ cells, as well as reduced infiltration of AQP5+ cells. Moreover, the numbers of caspase 3+ cells, a hallmark of apoptosis, increased. Thus, even with significantly fewer PBMCs than in previous models, our humanized NSG mouse model offers a valuable and efficient preclinical platform for investigating the early stage immunopathogenesis of SS. Chemokines CXCL9 and CXCL10 are ligands of CXCR3, promoting the recruitment of CXCR3-expressing cells to inflammatory sites and thereby contributing to SS progression (26). Elevated serum levels of CXCR3 and CXCL10 have been reported in patients with primary SS compared with healthy individuals (27), and increased CXCL9 expression in minor salivary glands has been associated with clinical disease severity (28). Furthermore, blockade of CXCR3 in NOD mice prior to disease onset improved salivary secretion and attenuated disease progression (29). These findings indicate that CXCR3-ligand interactions play a pivotal role in SS pathogenesis. In our study, the humanized SS model demonstrated significant upregulation of CXCL9 and CXCL10 directly within the salivary gland tissue (**Figure 3**). Crucially, we confirmed the local infiltration and distribution of CXCR3+ Th17 cells within these inflamed salivary glands using multi-color immunofluorescence (**Figures 4**, **7**). This local presence of pathogenic Th17 cells, paired with the high expression of their cognate chemoattractants (CXCL9/10), provides direct evidence of the CXCL9/10-CXCR3-mediated recruitment axis driving glandular destruction. Collectively, these findings demonstrate that our humanized SS model faithfully recapitulates the key the immunopathological mechanisms of the target organ in SS patients.

In our previous study, we demonstrated that metformin alleviated salivary gland inflammation in a murine SS model (22). Consistent with this, metformin administration in the current humanized SS model effectively ameliorated key pathological features, including inflammation and tissue damage. These findings further validate the therapeutic potential of metformin and demonstrate that the humanized SS mouse model reflects key aspects of the immune environment of SS patients. Metformin was selected as a key therapeutic agent in this study due to its potent immunometabolic modulatory effects, in addition to its traditional role in glucose metabolism. Pathogenic Th17 cells rely heavily on aerobic glycolysis for their pro-inflammatory functions; metformin-mediated AMPK activation inhibits these metabolic shifts, thereby suppressing Th17 differentiation and the CXCL9/10-CXCR3 recruitment. Our findings (**Figure 6**) demonstrating that metformin preserves AQP5 expression and inhibits Caspase-3 activation suggest that it acts as a disease-modifying agent that directly enhances the structural integrity of salivary epithelial cells against immune-mediated metabolic stress. The metformin dose used (50 mg/kg) was selected based on previous literature (22) observing the efficacy and safety of metformin in a mouse preclinical model. Due to the limited availability of patient-derived PBMCs and the technical complexity of the humanized NSG model, in-depth mechanistic analysis was prioritized over dose-response studies. Based on its two mechanisms of metabolic reprogramming of Th17 cells and protection of acinar cells, metformin has the potential to be a candidate for novel adjuvant therapy targeting early-stage immunometabolic dysfunction in SS. Particularly for patients who show an insufficient response to existing immunosuppressants such as hydroxychloroquine, metformin can provide a complementary approach by targeting the immunometabolic dysfunction underlying the disease.

In conclusion, by stimulating PBMCs from SS patients through T-cell receptor activation and transplanting a very small number of these cells (1-2 × 10^6^ cells) into NSG mice, we effectively recapitulated the hallmark immunopathological features of SS, particularly at the cellular and molecular levels. This model exhibited pronounced inflammation and functional impairment in salivary gland tissue, along with activation of the CXCL9/CXCL10–CXCR3 axis. As shown in **Supplementary Figure 1**, although the SFR exhibited a declining trend in all groups by week 5, no statistically significant difference was observed between the vehicle-treated group and healthy control group. We acknowledge the limitation of this study regarding the lack of direct functional phenotypes, such as measured salivary flow rates. While the 5-week observation period was optimized to capture the peaks of cell infiltration and molecular damage (e.g., AQP5 reduction and Caspase-3 activation), it may be insufficient for gross physiological changes in secretions to manifest fully, considering that molecular damage often precedes measurable secretory decline. Consequently, our findings should be interpreted as reflecting the early- stage pathogenic phase of salivary gland dysfunction. However, the moderated preservation of SFR in the metformin-treated group, aligned with the molecular preservation of AQP5, highlights the potential of metformin to delay functional decline at an early stage. Future studies incorporating longitudinal tracking or gland specific antigen challenges will be necessary to further validate the functional aspects of this platform. Overall, this humanized SS mouse model represents a valuable preclinical platform for evaluating therapeutic agents and identifying personalized treatments that reflect patient-specific immune characteristics.

## Data Availability

The original contributions presented in the study are included in the article/**Supplementary Material**. Further inquiries can be directed to the corresponding authors.
