# Supplementary material for: Development of an improved preclinical humanized mouse platform representing the diverse clinical phenotypes of Sjögren’s syndrome
Source: Front Immunol. 2026 May 22;17:1793493. doi: 10.3389/fimmu.2026.1793493 (PMC13236660; doi:10.3389/fimmu.2026.1793493)
Supplement: Supplementary file 1 [file DataSheet1.docx]

**Supplementary Figure 1.**


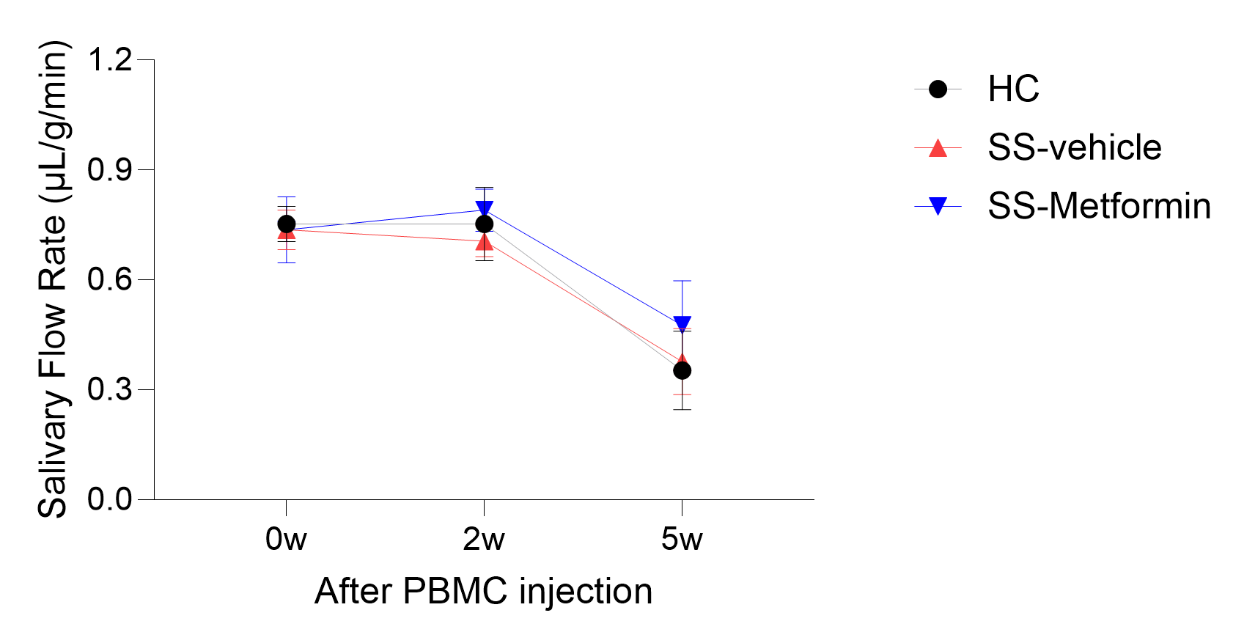


**Supplementary Figure 1.** Longitudinal assessment of salivary flow rate (SFR) in humanized NSG mice. SFR was measured at 0, 2, and 5 weeks after PBMC injection. Mice were anesthetized and saliva secretion was stimulated by intraperitoneal injection of pilocarpine (5 mg/kg). Saliva was collected from the oral cavity for 7 minutes, starting 90 seconds post-injection, using a micropipette. The volume of saliva was determined gravimetrically and normalized to body weight (μL/g/min). Data are presented as mean ± SEM.
