# Supplementary material for: Development of an improved preclinical humanized mouse platform representing the diverse clinical phenotypes of Sjögren’s syndrome
Source: Front Immunol. 2026 May 22;17:1793493. doi: 10.3389/fimmu.2026.1793493 (PMC13236660; doi:10.3389/fimmu.2026.1793493)
Supplement: Supplementary file 2 [file Table1.docx]

**Supplementary Table 1. Clinical Profiles of SS Patients and Healthy Controls**

| **Group** | **ID** | **Age** | **Sex** | **Disease duration (months)** | **Anti-Ro Ab positivity** | **Anti-La Ab positivity** | **Extra-glandular manifestation** | **ESSDAI score** |
| --- | --- | --- | --- | --- | --- | --- | --- | --- |
| **SS** | Patient 1 | 60 | F | 7 | Yes | No | Autoimmune thyroid disease | 4 |
|  | Patient 2 | 57 | F | 199 | No | No | Arthralgia/arthritis,  Lymphadenopathy | 4 |
|  | Patient 3 | 60 | F | 22 | Yes | Yes | Lymphadenopathy,  Cutaneous vasculitis | 15 |
|  | Patient 4 | 41 | F | 122 | Yes | Yes | Lymphoma | 0 |
|  | Patient 5 | 29 | F | 16 | Yes | Yes | Lymphadenopathy | 8 |
|  | Patient 6 | 39 | F | 90 | Yes | Yes | Lymphadenopathy,  Peripheral neuropathy | 11 |
| **HC** | Control 1 | 43 | F | N/A | Negative | Negative | None | 0 |
|  | Control 2 | 46 | F | N/A | Negative | Negative | None | 0 |
|  | Control 3 | 33 | F | N/A | Negative | Negative | None | 0 |
|  | Control 4 | 27 | F | N/A | Negative | Negative | None | 0 |
|  | Control 5 | 51 | F | N/A | Negative | Negative | None | 0 |

Abbreviations: SS, Sjögren’s syndrome; HC, healthy control; Ab, antibody; F, female; N/A, not applicable; ESSDAI, EULAR Sjögren's Syndrome Disease Activity Index.
